# Impact of the Adjunctive Use of Omega-3 in Periodontitis Patients With Diabetes on Local and Systemic Chemerin Levels: A Randomized Clinical Trial

**DOI:** 10.1155/ijod/7927250

**Published:** 2025-07-30

**Authors:** Dalia Ghalwash, Ahmed Ammar, Ahmed Hamdy, Ayman El-Gawish

**Affiliations:** Lecturer of Oral Medicine and Periodontology, Faculty of Dentistry, The British University in Egypt, El Sherouk City, Egypt

**Keywords:** cardiovascular risk, chemerin, diabetes, omega-3, periodontal therapy, periodontitis

## Abstract

**Aims:** The present study aspired to evaluate the impact of the adjunctive use of omega-3 with nonsurgical periodontal therapy on clinical parameters as well as local and systemic chemerin levels as a marker of cardiovascular disease risk in periodontitis patients with diabetes.

**Methods:** This randomized clinical trial was performed on thirty periodontitis patients with type II diabetes divided into two equal groups, both treated by nonsurgical periodontal treatment with the adjunctive use of daily 1000 mg Omega-3FAs in group I only for 6 months. Patients were reexamined after 2 weeks (baseline), 3, and 6 months for recording the clinical parameters as follows: plaque index (PI), gingival index (GI), probing depth (PD), and clinical attachment loss (CAL). Chemerin levels were assessed in both serum and GCF samples, HbA1c levels were also assessed.

**Results:** Omega-3FAs-treated group recorded a more statistically significant improvement in clinical parameters compared to the control group, particularly concerning PD and CAL. A statistically significant reduction of HbA1c levels between baseline, 3 and 6 m values was encountered in Omega-3FAs treated group, while no significant difference was evident in the control group. Additionally, Omega-3FAs treated group recorded a more statistically significant reduction of GCF and serum chemerin levels in comparison to the control group after 6 months of therapy.

**Conclusion:** The adjunctive use of omega-3FAs with nonsurgical periodontal therapy has resulted in significant improvement of clinical periodontal parameters and glycemic control in periodontitis patients with type II diabetes, alongside the additional benefit of reducing both local and systemic chemerin levels, a biomarker for cardiovascular risk.

**Trial Registration:** Clinical Trial Registry identifier: NCT06463535

**Summary**

• **Background:** The current investigation revealed the value of host modulation therapy with Omega-3FAs oral administration as an adjunct to nonsurgical periodontal therapy in the management of patients with periodontitis and type II diabetes.
• **Added value of this study:** The main strength of the present study is the assessment of both local and systemic chemerin levels allowing for better elucidation of the systemic effects of the therapeutic use of Omega-3FAs, which showed simultaneous reduction in both GCF and serum chemerin levels, a marker of systemic inflammation and cardiovascular risk, together with improvement of clinical parameters and glycemic control in those patients.
• **Clinical implications:** Development of new treatment strategies targeting the reduction of chemerin levels might have the potential to reduce both local and systemic inflammatory burden as well as cardiovascular risk in periodontitis patients, and Omega-3FAs is a promising and successful therapeutic candidate.

## 1. Introduction

Periodontitis is characterized by a hyperreactive inflammatory reaction in periodontal tissues involving neutrophils, macrophages, T and B cells, complement, cytokine networks, metalloproteinases, and lipid mediators, which contribute to tissue destruction and microvascular damage, with release of proinflammatory mediators into both local and systemic circulation [1, 2]. Local inflammation leading to alveolar bone breakdown in periodontitis is associated with low-grade inflammation (LGI) as is the case for cardiovascular diseases (CVDs), obesity, and diabetes [3]. Therefore, periodontal inflammation is assumed to be causally linked with the occurrence and progression of chronic systemic conditions through initiation of LGI, which is believed to be a silent risk factor for many systemic diseases [3].

Periodontitis and diabetes are chronic inflammatory diseases having common pathogenic mechanisms involving various proinflammatory mediators [4]. The uptake of advanced glycation end products during periodontitis provokes proinflammatory mediators leading to activation of VCAM-1 (vascular cell adhesion molecule 1), hyper-permeability of the endothelial cell, and the systemic release of TNF-*α*, IL-1, and IL-6, which are able to significantly modify endothelial function [3]. Such vascular inflammation is common in both periodontitis and in CVDs the major universal cause of global mortality [5, 6].

Additionally, periodontitis has been associated with a number of risk factors for CVD, including hypertension, diabetes, dyslipidemia, obesity, low socioeconomic status, depression, fatty liver, as well as with other chronic inflammatory diseases as rheumatoid arthritis, psoriasis, and inflammatory bowel disease [7]. Furthermore, evidence has implied that periodontitis and CVD have shared genetic susceptibilities, particularly coronary artery disease [8]. It was also reported that periodontal inflammation complicates the initial atherosclerotic processes and speeds up the formation of atherosclerotic plaque [9]. Moreover, oxidative stress and inflammatory pathways have been implicated in the link between periodontitis and myocardial infarction, which is further confirmed by the upregulation of certain important antioxidant enzymes in periodontitis [10, 11].

Recent interventional studies have revealed a reduction in circulating levels of inflammatory biomarkers after period in therapy with a potential subsequent advantage of reducing cardiovascular risk as improving endothelial dysfunction by lowering LGI which would be reflected by reductions in systemic levels of biomarkers, particularly in patients with established diabetes, CVD, or both [3, 12]. Periodontal therapy has also been related to a moderate decrease of hemoglobin A1c (HbA1c) in diabetic patients [5].

Omega-3 polyunsaturated fatty acids (Omega-3FAs) including eicosapentaenoic acid (EPA) and docosahexaenoic acid (DHA), have been recently investigated for their various roles in health promotion and disease risk reduction including, rheumatoid arthritis, cardiovascular disease, atherosclerosis, fatty liver disease, diabetes, and periodontitis [4, 13]. The beneficial role of Omega-3FAs in the management of such chronic inflammatory and autoimmune diseases are mainly attributed to the anti-inflammatory, vasodilator, antithrombotic, anti-arrhythmic, and hypolipidemic effects [14]. Omega-3FAs consumption has been reported to significantly reduce triglycerides and interfere with mechanisms of atherosclerosis that results in reduced cardiovascular mortality and morbidity [15]. Moreover, a large meta-analysis projected that daily Omega-3FAs supplementation is associated with lower risk of myocardial infarction, coronary heart disease death, total coronary heart disease, cardiovascular disease death, and total cardiovascular disease [16].

Chemerin is a multifunctional adipokine that has recently been acknowledged to play fundamental roles in the pathogenesis of hypertension, diabetes, preterm birth, inflammatory diseases, metabolic disorders, liver cirrhosis, and CVDs [17]. Chemerin plays an important part in the immune response as it acts as a chemoattractant for various immune cells, besides its role in regulating adipogenesis, energy metabolism, and angiogenesis [18]. Moreover, circulating chemerin levels have been reported to be positively correlated with the severity of coronary artery disease. Increased plasma chemerin levels are an independent predictor of coronary artery disease and are linked with higher risk of substantial adverse events in CVD patients [19]. Furthermore, chemerin levels are positively correlated with aortic and coronary atherosclerosis as well as peripheral arterial stiffness [20]. This relates to the effects of chemerin on the atherogenic process, involving vascular remodeling, lipid deposition, and inflammation [21].

Accordingly, the present study aimed to evaluate the effects of omega-3 as an adjunct to nonsurgical periodontal therapy on clinical periodontal parameters as well as gingival crevicular fluid (GCF) and serum chemerin levels as a marker of cardiovascular disease risk in periodontitis patients with diabetes.

## 2. Subjects and Methods

### 2.1. Sample Size Calculation

Based on research assessing the impact of omega-3 use in management of periodontal disease [22], by fixing alpha at 0.05 and beta at 0.2 the CAL improved in study from 5.1 to 3 mm in control after 6 months, the effective size f is 0.9. The minimal sample size to be included is 12 in each group to avoid attrition 20% was added, thus, 15 patients were included in each group.

### 2.2. Trial Design

A parallel design randomized clinical trial was performed involving 30 participants divided into two groups, 15 participants in each group selected from the outpatient clinic in Oral medicine and Periodontology department, faculty of Dentistry, The British University in Egypt during the period from September 2023 to April 2024. The Protocol of the study was clarified for each patient after that an informed consent was signed by each participant. The study was conducted in accordance with the Declaration of Helsinki on ethical principles for medical research involving human subjects. Ethical approval of the study was acquired from the Research Ethics Committee in the British University in Egypt.

### 2.3. Inclusion Criteria

Patients with periodontitis stage II to stage IV, age between 30 and 70 years, patients suffering from type 2 DM for more than 2 years, with no other systemic problem.

### 2.4. Exclusion Criteria

Pregnant and lactating females, patients with other autoimmune or systemic disease, smokers, or former smokers, patients who took antibiotics in the last 6 months and patients with no or poor adherence to our study protocol or plaque control instructions, patients not showing up for the 3-month or 6-month follow-ups.

### 2.5. Clinical Examination

Medical and dental history, extra and intra oral examination was done for these patients by (AE) to be sure that they fulfilled the inclusion and exclusion criteria of the study. A detailed medical history of each subject was acquired according to the modified Cornell Medical Index.

Glycemic control was assessed through measurement of glycosylated hemoglobin A1c (HbA1c) in blood samples of patients having relatively stable glycemic control, established when the difference between at least two HbA1c readings did not exceed 1% in the previous 6 months. HbA1c was assessed at baseline, 3 and 6 m.

### 2.6. Randomization

The patients were randomly assigned to group I (intervention) and group II (control) by generating a table including a random sequence of numbers created by Random Allocation Software1.

Patient grouping

• Group I (nonsurgical periodontal therapy + Omega-3FAs).
• Group II (nonsurgical periodontal therapy).

Periodontal treatment protocol

Group I and II patients were treated by nonsurgical approach by (A.A.) in following steps:

1. Each patient had received an initial phase of detailed instruction in self-performed plaque control measures using soft toothbrush and interdental cleansing devices.
2. Full mouth nonsurgical periodontal therapy using ultrasonic scaler and hand instruments was performed to each patient in two sessions.
3. Chlorehexidine mouthwash was prescribed for patients to be used twice daily.
4. For each patient, follow up visits every 2 weeks were done to ensure plaque control.
5. Patients were reexamined after 2 weeks (baseline), 3, and 6 months for recording the different clinical parameters; plaque index (PI), gingival index (GI), probing depth (PD), and clinical attachment loss (CAL).
6. Omega-3FAs (1000 mg) was given as an adjunctive treatment daily for 6 months to group I, starting 2 weeks after phase 1 therapy.

### 2.7. Serum and GCF Sampling

Serum and GCF samples were collected at baseline 3 and 6 months from all participants. The samples were collected on the day after the patients had their periodontal assessment and diagnosis.

For individuals with healthy periodontium, GCF samples were acquired from areas which showed almost no clinical inflammation (PI and GI = 0 or 1). For periodontitis patients, GCF samples were collected from the sites with the most obvious signs of clinical inflammation and deepest PD with radiographic evidence of bone loss.

GCF samples at the selected sites were collected as follows: all the supragingival plaque must be removed gently removed with sterile cotton roll, the tooth air dried gently, and isolated with sterile cotton roll to avoid contamination with saliva. Paper strips are then placed gently into the sulcus or pocket until minimal resistance is felt and lest for 30 s. Any strip contaminated with blood or saliva was discarded.

Serum and GCF samples from each subject were then placed into sterile Eppendorf container and stored immediately at −80°C until the biochemical analysis.

### 2.8. Biochemical Analysis

200 µL of phosphate buffer saline was added to the paper strip in the Eppendorf then vortex was done followed by centrifugation for 10 min at 3000 Xg. The supernatant was used for estimation of chemerin level.

The GCF and serum chemerin levels were measured using the RD191136200R Human Chemerin ELISA2. Samples are sheltered in microwells coated with polyclonal antihuman chemerin antibody (PCA), and incubated for 60 min then washed, followed by the addition of biotin labeled PCA and incubated for another 60 min and washed again. Then incubated with streptavidin-HRP conjugate for 30 min before the final washing step, the residual conjugate is permitted to react with the substrate solution. Then an acidic solution is added, and absorbance of the residual yellow product is assessed, which is proportional to chemerin concentration.

### 2.9. Statistical Analysis

The data were explored for normality using Kolmogorov–Smirnov and Shapiro Wilk tests, data showed parametric (normal) distribution. Independent sample *t*-test was used to compare between two groups in nonrelated samples. Repeated measure ANOVA was used to compare between more than two groups in related samples. A paired sample *t*-test was used to compare between two groups in related samples. For nonparametric data; Mann Whitney test was used to compare between two groups in nonrelated samples Friedman test was used to compare between more than two groups in related samples. The Wilcoxon test was used to compare two groups in related samples. The significance level was set at *p* ≤ 0.05. Statistical analysis was performed with IBM SPSS Statistics Version 20 for Windows.

## 3. Results

### 3.1. Study Population and Demographic Data

**Figure 1:**
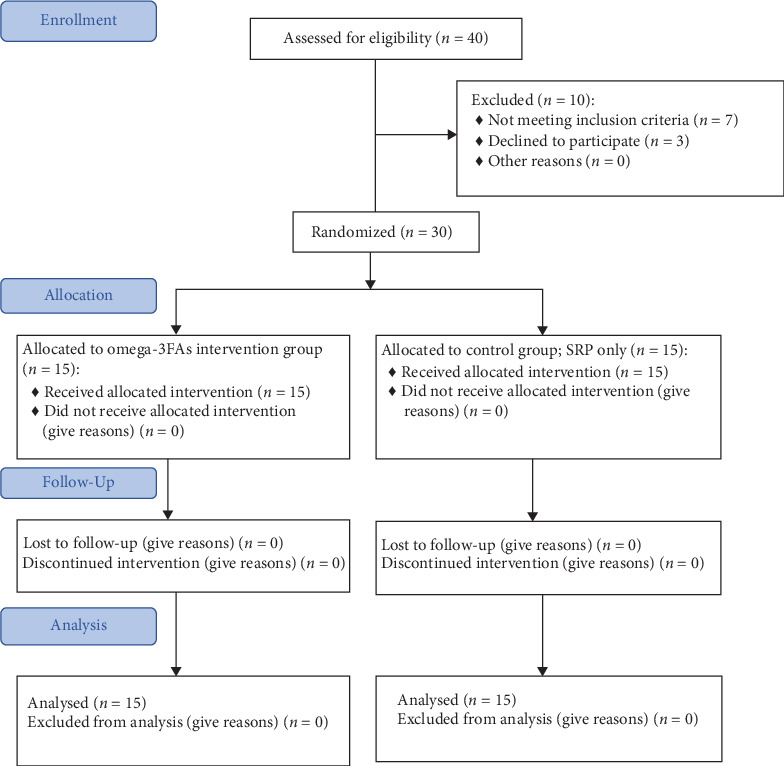
Flow diagram of the study participants.

As presented in the flow diagram in Figure 1 a total of 30 individuals participated in the present study divided into two groups; Omega-3FAs + nonsurgical periodontal therapy intervention group composed of 15 patients (six males and nine females) with mean age of 40.4 years, and the control group (nonsurgical periodontal therapy only) composed of 15 patients (6 male and 9 females) with mean age of 38.47. Both gender and age range were closely matching in both groups.

### 3.2. Clinical Parameters

#### 3.2.1. PI

There was a statistically significant difference between baseline, 3 and 6 m in group I (*p*  < 0.001) and in group II (*p*=0.001). No statistically significant difference was found between group I and group II at all time intervals.

#### 3.2.2. GI

A statistically significant difference was found between baseline, 3 and 6 m in group I (*p*  < 0.001) and in group II (*p*=0.012). No statistically significant difference was found between group I and group II at all time intervals.

#### 3.2.3. PPD

For group I there is a statistically significant difference between all-time intervals. There was a statistically significant difference between baseline, 3 and 6 m where (*p*  < 0.001), and between 6 m and each of baseline and 3 m in group II. The only statistically significant difference between group I and group II were encountered at 3 m interval (*p*=0.011) Table 1.

#### 3.2.4. CAL

A statistically significant difference was found between all time intervals in both groups I and II. While a statistically significant difference between Group I and Group II was encountered at 3 and 6 m intervals (*p*  < 0.001) Table 2.

### 3.3. Chemerin Serum Levels

For both Group I and II; a statistically significant difference was found between all-time intervals (*p*  < 0.001). The only significant difference between group I and II was found at 6 m where (*p*  < 0.001) Table 3.

### 3.4. Chemerin GCF Levels

A significant difference between each time interval (*p*  < 0.001) was evident in both groups. The only significant difference between group I and II was found at 6 m where (*p*  < 0.001) Table 4.

### 3.5. HgA1c

For group I, a statistically significant difference was found between baseline and each of 3 and 6 m and between 3 and 6 m where (*p*  < 0.001). While no significant difference was recorded in group II Table 5.

A statistically significant difference between group I and group II were found at 3 and 6 m (*p*=0.001) and (*p*  < 0.001), respectively.

## 4. Discussion

The result of the present study showed that all clinical parameters including PI, GI, PD, and CAL in both groups have been improved 3 and 6 months after nonsurgical periodontal therapy. However, Omega-3FAs treated group yielded a more statistically significant improvement in clinical parameters in comparison to group II (the control group) particularly concerning PD and CAL.

Such results are in accordance with results of a recent systematic review and meta-analysis concluding that the adjunctive use of Omega-3FAs has led to statistically significant CAL gain and PD reduction when compared to nonsurgical periodontal therapy alone [14]. Similar results were also conveyed by an earlier systematic review that evaluated the impact of the adjunctive use of Omega-3FAs in the treatment of periodontitis [23].

However, another recent systematic review could not reach a satisfactory conclusion regarding the benefits of Omega-3FAs supplement for periodontal treatment probably due to inconsistency in the methodology or discrepancy in the obtained findings and the elevated bias risk [24].

The beneficial effects of adjunctive use of Omega-3FAs might be attributed to the production of bioactive lipid mediators as resolvins and protectins with protective, immunoregulatory and anti-inflammatory effects, and inhibition of chemotaxis and proinflammatory mediators [22]. Moreover, Omega-3FAs inhibit the secretion of chemerin from adipocytes [25]. This inhibition might contribute to the anti-inflammatory effects of Omega-3FAs [26]. Furthermore, EPA and DHA have antibacterial actions and can inhibit the activity of some periodontal pathogens, such as *Porphyromonas gingivalis*, *Prevotella intermedia*, and *Fusobacterium nucleatum* [27 ].

HbA1c is the gold standard parameter for assessing glycemic control and clinical management of diabetic patients as it reflects serum glucose levels during the last 3 months [4]. In the current research, HbA1c levels were estimated at baseline, 3 and 6 m after treatment protocol to evaluate its impact on glycemic control, where a statistically significant reduction of HbA1c levels between baseline, 3 and 6 m values in Omega-3FAs treated group was encountered, while no significant difference was evident in the control group. In line with these results, other studies reported a nonsignificant difference in HbA1c after nonsurgical periodontal therapy [28, 29]. On the other hand, nonsurgical therapy was reported to be effective in improving the glycemic control in type 2 diabetes mellitus patients [5, 30].

Omega-3FAs group also registered a statistically significant reduction in HgA1c levels in comparison to the control group after 3 and 6 m of therapy. Indicating a significant improvement in glycemic control in Omega-3FAs treated patients which is in accordance with previous research [4, 22, 31].

The reduction in HbA1c levels after adjunctive use of Omega-3FAs to nonsurgical periodontal therapy is attributed to the reduction of inflammatory burden resulting from combined effect of periodontal therapy and Omega-3FAs leading to down regulation of several proinflammatory markers. Which agrees with research revealing an association between glycemic control and periodontitis through systemic inflammation [32]. Studies on numerous proinflammatory cytokines have shown that cytokines are regulated by complicated signaling pathways that mediate both diabetes and periodontal disease pathogenesis. One such cytokine that attained more focus recently is an important adipokine namely chemerin which is involved in both diabetes and periodontal diseases because of its considerable effects on insulin sensitivity, glucose levels, lipid metabolism, and inflammatory disease process [17]. Chemerin is chemoattractant for leukocytes, toward areas of inflammation, and supports linking macrophages to extracellular matrix proteins, adhesion molecules, and tissue endothelium [18]. Chemerin possesses a proinflammatory influence and its levels have been reported to correlate positively with recognized markers of inflammation, such as IL-6, TNF-*α*, and C-reactive protein [33]. Additionally, chemerin levels have been reported to correlate with severity of periodontal inflammation and are considered prospective biomarkers of inflammation in periodontitis as well as type II diabetes [34].

In the current investigation, a statistically significant decrease in GCF and serum chemerin levels with time was noted in both groups. This is in accordance with recent research reporting a similar reduction in chemerin levels after nonsurgical periodontal therapy [35]. While Omega-3FAs treated group recorded a more significant reduction of GCF and serum chemerin levels in comparison to the control group after 6 months of therapy. This reduction in both GCF and serum chemerin levels being a proinflammatory marker is mainly attributed to the removal of etiological factors and reduction of inflammatory component in the periodontal tissues through nonsurgical periodontal therapy which is further augmented by the adjunctive use of Omega-3FAs, demonstrating that periodontal diseases possess systemic impacts continuing beyond the local periodontal environment. This is in accordance with research reporting that periodontal therapy improves systemic inflammation in diabetic patients [4, 5, 31, 35].

The persistent inflammation occurring in periodontitis is commonly associated with an amplified cardiovascular risk, with systemic inflammation and bacteremia possibly initiating endothelial dysfunction with potentiation of vascular inflammation, which is modulated by proinflammatory cytokines including adipokines as chemerin, in addition to IL-1, IL-6, and TNF-*α*, both in chronic periodontitis and in CVDs [36]. Additionally, several research showed that periodontitis can induce systemic inflammation with exacerbation of atherosclerotic lesions [36, 37]. It has been reported that a reduction in levels of circulating inflammatory biomarkers following periodontal therapy contributes to reduction of cardiovascular risk [5, 36]. Thus, periodontal therapy with adjunctive use of Omega-3FAs in diabetic patients could lead to reduction of cardiovascular disease risk through reduction of systemic inflammation.

Chemerin levels are increased in some chronic inflammatory diseases and are mainly associated with disease severity [35]. An association between chemerin levels and cardiovascular disease has been revealed in several research, as serum chemerin levels were correlated with atrial fibrillation, dilated cardiomyopathy, and cardiovascular disease severity [20, 38, 39]. Additionally, high chemerin levels were found to be predictive of cardiovascular events [40]. Furthermore, recent research revealed that high chemerin levels were considered an independent predictor of coronary artery disease, and a significant and independent predictor of major adverse cardiovascular events in coronary artery disease patients and proposed using chemerin as a novel biomarker for the early diagnosis and prognosis of CVDs [19].

Omega-3FAs therapy has a role in reducing cardiovascular risk possibly through the reversal of endothelial cell dysfunction which is causally related to atherosclerosis and is associated with increased cardiovascular risk [15]. Another possibility proposed by the current investigation is reducing cardiovascular risk through reducing chemerin level, the adipokine closely correlated with cardiovascular disease severity.

A limiting factor in the current study is the limited sample size and the liability of many patients for not adhering firmly to treatment protocol probably due to the long follow up period leading to rejection of some patients to participate in the trial or loss of some patients during the follow up period which necessitates their replacement with similar patients. Selection of participants having common conditions with reasonable selection criteria in addition to choosing interventions that are feasible to apply were carried out in the present study to ensure the external validity and the generalizability of our results. Additionally, a main strength point in the present study is the assessment of both local and systemic chemerin levels allowing for better elucidation of the systemic effects of the therapeutic use of Omega-3FAs in conjunction with nonsurgical periodontal therapy in patients with periodontitis and type II diabetes mellitus. Which showed simultaneous reduction in both GCF and serum chemerin levels, a marker of systemic inflammation and cardiovascular risk, together with improvement of clinical parameters and glycemic control in those patients. Thus, developing new treatment strategies targeting chemerin levels might have the potential to reduce both local and systemic inflammatory burden as well as cardiovascular risk in periodontitis patients, and Omega-3FAs is a promising and successful candidate.

## 5. Conclusion

The current investigation revealed the value of host modulation therapy with Omega-3FAs oral administration as adjunct to nonsurgical periodontal therapy in management of patients with periodontitis and type II diabetes, as it resulted in significant improvement of clinical periodontal parameters and glycemic control with the additional benefit of reducing both local and systemic chemerin levels a biomarker for cardiovascular risk.

## Figures and Tables

**Table 1:** The mean, standard deviation (SD) values of PD of different groups.

Variables	PD								
	Group I				Group II				*p*-Value
	Mean	SD	Min	Max	Mean	SD	Min	Max	
Baseline	6.07^a,A^	0.80	5.00	7.00	6.07^a,A^	0.88	5.00	8.00	**1 ns**
After 3 m	5.07^b,B^	0.80	4.00	6.00	5.80^a,A^	0.68	5.00	7.00	**0.011*⁣*^*∗*^**
After 6 m	4.40^c,A^	0.83	3.00	6.00	5.07^b,A^	1.03	4.00	8.00	**0.061 ns**
*p*-Value	**<0.001*⁣*^*∗*^**				**<0.001*⁣*^*∗*^**				—

*Note*: Means with different small letters in the same column indicate statistically significance difference. Means with different capital letters in the same row indicate statistically significance difference. The statistical results as well as the reference value are made in bold. *⁣*^*∗*^, significant (*p*  < 0.05); ns, nonsignificant (*p*  > 0.05).

**Table 2:** The mean, standard deviation (SD) values of CAL of different groups.

Variables	CAL								
	Group I				Group II				*p*-Value
	Mean	SD	Min	Max	Mean	SD	Min	Max	
Baseline	6.40^a,A^	1.24	5.00	9.00	6.67^a,A^	0.82	5.00	8.00	**0.493 ns**
After 3 m	4.47^b,B^	1.06	3.00	7.00	6.00^b,A^	0.65	5.00	7.00	**<0.001*⁣*^*∗*^**
After 6 m	3.40^c,B^	0.74	2.00	5.00	5.40^c,A^	0.74	4.00	7.00	**<0.001*⁣*^*∗*^**
*p*-Value	**<0.001*⁣*^*∗*^**				**<0.001*⁣*^*∗*^**				—

*Note*: Means with different small letters in the same column indicate statistically significance difference. Means with different capital letters in the same row indicate statistically significance difference. The statistical results as well as the reference value are made in bold. *⁣*^*∗*^, significant (*p*  < 0.05); ns, nonsignificant (*p*  > 0.05).

**Table 3:** The mean, standard deviation (SD) values of Chemerin serum of different groups.

Variabes	Chemerin serum								
	Group I				Group II				*p*-Value
	Mean	SD	Min	Max	Mean	SD	Min	Max	
Baseline	37.47^a,A^	6.42	28.50	50.20	35.25^a,A^	5.13	27.50	43.10	**0.304 ns**
After 3 m	24.99^b,A^	4.27	19.10	32.30	27.59^b,A^	3.48	21.20	32.30	**0.078 ns**
After 6 m	16.33^c,B^	2.87	12.10	21.30	22.6^c,A^	3.30	18.20	28.30	**<0.001*⁣*^*∗*^**
*p*-Value	**<0.001*⁣*^*∗*^**				**<0.001*⁣*^*∗*^**				—

*Note*: Means with different small letters in the same column indicate statistically significance difference. Means with different capital letters in the same row indicate statistically significance difference. The statistical results as well as the reference value are made in bold. *⁣*^*∗*^, significant (*p*  < 0.05); ns, nonsignificant (*p*  > 0.05).

**Table 4:** The mean, standard deviation (SD) values of Chemerin GCF of different groups.

Variables	Chemerin GCF								
	Group I				Group II				*p*-Value
	Mean	SD	Min	Max	Mean	SD	Min	Max	
Baseline	204.97^a,A^	28.88	164.10	235.20	187.60^a,A^	43.36	133.20	265.20	**0.207 ns**
After 3 m	149.09^b,A^	19.59	119.30	178.20	154.23^b,A^	26.75	117.30	201.40	**0.553 ns**
After 6 m	93.99^c,B^	10.75	73.50	109.40	126.30^c,A^	20.84	99.20	173.50	**<0.001*⁣*^*∗*^**
*p*-Value	**<0.001*⁣*^*∗*^**				**<0.001*⁣*^*∗*^**				—

*Note*: Means with different small letters in the same column indicate statistically significance difference. Means with different capital letters in the same row indicate statistically significance difference. The statistical results as well as the reference value are made in bold. *⁣*^*∗*^, significant (*p*  < 0.05); ns, nonsignificant (*p*  > 0.05).

**Table 5:** The mean, standard deviation (SD) values of HgA1c of different groups.

Variables	HgA1c								
	Group I				Group II				*p*-Value
	Mean	SD	Min	Max	Mean	SD	Min	Max	
Baseline	8.59^a,A^	0.59	7.80	9.90	8.53^a,A^	0.89	7.30	10.10	**0.830 ns**
After 3 m	7.56^b,B^	0.44	7.00	8.50	8.39^a,A^	0.69	7.60	9.80	**0.001*⁣*^*∗*^**
After 6 m	6.49^c,B^	0.31	6.10	7.30	8.12^a,A^	0.63	7.30	9.50	**<0.001*⁣*^*∗*^**
*p*-Value	**<0.001*⁣*^*∗*^**				**0.099 ns**				—

*Note*: Means with different small letters in the same column indicate statistically significance difference. Means with different capital letters in the same row indicate statistically significance difference. The statistical results as well as the reference value are made in bold. *⁣*^*∗*^, significant (*p*  < 0.05); ns, nonsignificant (*p*  > 0.05).

## Data Availability

The data that support the findings of this study are available from the corresponding author upon reasonable request.

## Nomenclature

Omega-3FAs: Omega-3 polyunsaturated fatty acids
EPA: Eicosapentaenoic acid
DHA: Docosahexaenoic acid
CVDs: Cardiovascular diseases
GCF: Gingival crevicular fluid
PI: Plaque index
GI: Gingival index
PD: Probing depth
CAL: Clinical attachment loss.

## Endnotes

^1^Random Allocation Software version 1.0. (Mahmood Saghaei, Iran).

^2^BioVendor – Laboratorní medicína, Guang Zhou, China.
